# NETosis in cancer

**DOI:** 10.18632/oncoscience.264

**Published:** 2015-11-16

**Authors:** Jessica Cedervall, Anna-Karin Olsson

**Affiliations:** Department of Medical Biochemistry and Microbiology, Science for Life Laboratory, Uppsala University, Biomedical Center, Uppsala, Sweden

**Keywords:** Neutrophil Extracellular Traps, NETs, cancer, vascular function, systemic inflammation

A large proportion of cancer-related deaths are caused by thrombosis and general organ failure. Although the awareness of tumor-induced systemic effects has increased significantly in recent years, current knowledge is still mainly restricted to metastatic sites. Surprisingly little is known about the situation in organs that are not targets for metastasis or directly affected by the primary tumor. We therefore decided to look deeper into this relatively unexplored field of cancer research. For obvious reasons human biopsy material from tissues not affected by tumor cells, in an individual with cancer, are rare and mouse models therefore become important tools for such investigations. Using two different orthotopic and spontaneously metastasizing tumor models - the RIP1-Tag2 model for insulinoma with metastasis to the liver and the MMTV-PyMT model for mammary carcinoma with lung metastasis - we analyzed the presence of hematopoietic cells in organs which do not represent sites for primary tumor growth. There was a significant increase in the number of neutrophils in heart and kidneys of tumor-bearing mice compared to healthy individuals [1]. In mice with cancer, peripheral organs displayed systemic inflammation and impaired vascular function, which was restored upon neutrophil depletion. Platelet/neutrophil complexes, indicative of neutrophil extracellular traps (NETs), were found in kidney and heart from tumor-bearing mice, while these complexes were completely absent in the corresponding tissues from healthy mice. Indeed, analysis of peripheral blood confirmed the presence of neutrophils with extracellular DNA-tails in tumor-bearing mice.

NETs were identified in 2004 and are extracellular networks that primarily consist of DNA released from neutrophils together with antimicrobial peptides and proteases derived from neutrophil granules. Furthermore, platelets aggregate to the NETs due to the procoagulant effect exerted by the negatively charged chromatin. These NETs trap and kill bacteria and were identified as a novel mechanism by which the innate immune system protects us from infections, especially in situations with sepsis. Over the last years it has however become increasingly clear that NETs also can form under non-infectious inflammatory conditions, like thrombosis, cancer, SLE, atherosclerosis and diabetes, and that the NETs cause damage to the endothelium [2-4]. NETosis may provide a mechanistic explanation for the risk of thrombosis during infection and inflammation and possibly also for cancer-induced deep-vein thrombosis.

Due to their high content of extracellular DNA, NETs can be destabilized and degraded by DNase. Somewhat unexpected, the impaired peripheral vessel function that we observed in tumor-bearing mice was completely restored and inflammation was suppressed after systemic treatment with DNase. This finding strongly suggests that tumor-induced systemic and intravascular NET formation reduces vascular function in organs not directly affected by the primary tumor or metastases. It will be very interesting to analyze if the same mechanisms are active in humans with cancer and ultimately if a NET-inhibiting drug could prevent dysfunction of peripheral organs. One can speculate that this NET-induced inflammatory condition in peripheral organs in an individual with cancer could be a contributing factor to metastasis. In support of such a scenario we found an upregulated expression of adhesion molecules in the endothelium of kidneys from tumor bearing mice, which could possibly be used by tumor cells for extravasation into secondary sites. If this up-regulation of adhesion molecules is a general process occurring throughout the body in individuals with cancer, including sites where metastasis develops, remains to be explored. If so, early treatment of cancer patients with a NET-inhibiting drug could prove beneficial in preventing dissemination of tumor cells.

Protein-arginine deiminase 4 (PAD4) has been strongly implicated in the formation of NETs and more specifically in the histone citrullination that occurs during NETosis. Novel, selective PAD4 inhibitors have recently been described with the capacity to disrupt formation of both human and mouse NETs [5]. These small molecule inhibitors may provide a clinical tool to prevent unwanted NETosis. Considering the number of diseases where NETs are believed to play a role, the therapeutic potential of these drugs should be significant.

But do we need the NETs? Do we put ourselves at risk to life-threatening sepsis if we use NET-inhibiting drugs? Although it has been reported that PAD4−/− mice are more susceptible for infection [6], others report that bacteremia is unaffected in PAD4-deficient mice, highlighting the potential benefit of PAD4 inhibition in inflammatory or thrombotic diseases [7].

In conclusion, improved cancer therapy is urgently needed - not only for the purpose of eliminating tumor cells - but also to provide therapeutic intervention that prevents tumor-induced systemic effects from becoming irreversible in an individual with cancer. Targeting NETosis may provide such an opportunity.
